# A Novel Technique to Mitigate the Data Redundancy and to Improvise Network Lifetime Using Fuzzy Criminal Search Ebola Optimization for WMSN

**DOI:** 10.3390/s23042218

**Published:** 2023-02-16

**Authors:** M. A. Matheen, S. Sundar

**Affiliations:** School of Electronics Engineering, Vellore Institute of Technology, Vellore 632014, India

**Keywords:** wireless multimedia sensor network, data redundancy, criminal search, Ebola optimization, fuzzy and cluster head

## Abstract

Wireless Multimedia Sensor Network (WMSN) is a powerful technology that is widely used to gather data and monitor the actual environment for analysis. Furthermore, multimedia applications’ needs and the features, such as constrained latency and high bandwidth consumption, complicate the design of WMSN routing protocols. Despite several methods, the trouble of designing WMSNs routing protocol remains a hurdle. The miniaturization and enhancement of hardware facilitate an extensive range of applications in the military and public sectors. On the contrary, the streaming of multimedia content is captured and generated due to some event-triggered surveillance for a long duration of time. Hence, it is necessary for wireless multimedia sensor network (WMSN) to provide a strong hardware foundation, thereby satisfying Quality of Service (QoS) requirements. Initially, the network is clustered into several clusters and the nodes with rich resources are chosen as cluster heads. The significant intention of this paper is to eliminate data redundancy and to select optimal cluster heads, thereby minimizing the energy consumption. Therefore, this paper proposes a novel Fuzzy Criminal Search Ebola Optimization (FCSEO) algorithm for optimal selection of cluster heads. In addition to this, the data redundancy present in the proposed algorithm is mitigated and thus the network lifetime is enhanced. Finally, extensive experimentation is carried out for various performance measures to determine the efficiency of the proposed approach.

## 1. Introduction

With the wireless communication advancement technology and smart terminals’ widespread fame, the applications of mobile multimedia are essential tools for work and daily life. Mobile multimedia applications are becoming increasingly popular due to their extensive functionality, good experience, and ubiquitous access. However, mobile multimedia service providers acquire user personal information when providing services to boost user viscosity, enhance user experience, or reserve data resources. Users must accept the privacy disclosure risk while making use of the ease of mobile multimedia services [1]. For example, the online browser history will reveal political learning’s, individuals’ consumption patterns, and other private information [2].

The various application domains that use WMSNs are military, industry, healthcare, and defense [3]. Over the last year, several investigations on object tracking, environment detection, target detection, border surveillance, and enemy intrusion have been carried out [4]. Multimedia applications create significant network traffic volume, overheads, and minimizing energy consumption is a major challenge in WMSNs. This traffic requires processing skills and high transmission rates on the part of the nodes. As both of these functions require a lot of energy, they can cause depletion of premature energy nodes and as a result, the network lifespan is reduced. Hence, in WMSNs, a dependable routing protocol and the creation of an effect that increases the network lifespan while also fulfilling the service quality needs of multimedia applications are crucial [5]. The WMSNs’ intrinsic qualities, such as limited bandwidth, dynamic links, and variable channel capacity, pose significant challenges to the design of WMSN routing protocols. Also, the limited resources, namely processing capability, buffer sizes, memory, bandwidths, and energy, are all factors that add to the difficulty of designing routing algorithms for WMSNs [6,7]. To overcome the issue, creating a routing protocol that detects routes effectively and selects a route among the sink and the detecting nodes. The routing protocol that uses a single path approach is particularly vulnerable to the extremely dynamic nature of wireless networks, and the multi-hop route has a limited capacity, and they are unable to provide high data communication for WMSNs [8]. Furthermore, the sensor nodes’ resources are limited in terms of memory, computational energy, and bandwidth, and congestion can have a significant impact on WMSN performance [9,10]. Numerous research works based on energy efficient wireless multisensor routing protocols have been developed. Meanwhile, those techniques faced the above mentioned limitations, which motivated us to present a novel article. The significant intention of this paper is to eliminate data redundancy and thereby select the optimal cluster head (CH) and minimize the energy consumption as well as enhance the network lifetime.

The major contributions of the article are depicted below:

- To propose a novel fuzzy criminal search Ebola optimization (FCSEO) algorithm for the optimal selection of cluster heads.
- To mitigate data redundancy, thereby enhancing the network lifetime and minimizing the energy consumption.
- To conduct extensive experimentation between various approaches to determine the effectiveness of the proposed system.

The remaining section of the paper is structured as follows. In Section 2, the survey for the cluster head selection process based on energy efficient routing protocols is discussed. Section 3 discusses the network model and energy consumption model. The proposed approach based on data redundancy elimination as well as cluster head selection is presented in Section 4. The results for various parameters are presented in Section 5. At last, the conclusion of the article is presented in Section 6. 

## 2. Related Works

The enhancing of the resource-constrained IoT device lifetime that mitigates redundant transmission was illustrated by Rehman et al. [11]. The nodes were able to recover the videos, audios, and images in a WMSN. When the declaration process of the CH and cluster formation process were completed, then the sub-cluster (SC) formation process was automatically activated. The sub-cluster head (SCH) was used for collecting data from the member node, eliminating all redundancies, and transferring minimum data to its corresponding CH. The PE-WMoT selected the fuzzy approach to choose the perfect SCH among all SC. The simulation result showed the number of redundant packet transmission reductions, increased throughput, and energy consumption. The drawback of this method is that it is only applicable for low-speed applications.

Aswale et al. [12] elaborated the triangle link quality metrics and minimum inter-path interference geographic multipath routing protocol for a WMSN. This protocol was employed for eliminating all hidden node problems (HNP) without considering the handshake mechanism. The result showed the improvement of performance and lifetime of the network.

Genta et al. [13] established wireless multimedia sensor networks using an energy-effective multipath routing (EMR) algorithm for data communication to decrease energy consumption. Multimedia applications create heavy traffic in the network, which leads to the generation of high energy consumption. The result showed high performance and minimum energy consumption. The main drawback of this method was the same application was not suitable for mobile sensor nodes. 

Raja et al. [14] proposed a technique called firefly load balancing based energy optimized routing (FLB-EOR) in WMN. This FLB-EOR approach determines the node networks with minimum weightage for multimedia data transmission to improve load balancing efficiency (LBE) and throughput. To transmit the multimedia data from sender to receiver, the gravitational neighbor-node selection technique is utilized to search the shortest neighbor. Hence, this FLB-EOR technique can decrease EC and enhance the LBE of multimedia data delivery. 

Awad et al. [15] presented the framework with the use of Gaussian distribution in multisource multipath routing for delay node optimization in a WMSN. Some of the parameters of networks are communication range and total routing paths needed, and source nodes are used to determine the optimal SD (standard deviation) and the theoretical bare minimum of relay nodes that are required. An analysis is conducted to determine the mathematical design and estimate the performance of the mathematical design. A statistical study found that routing efficiency was at least doubled when using an optimal Gaussian distribution rather than the uniform distribution. In addition, the number of packet controls and the average path lengths are significantly reduced to enhance pocket delay, data distribution, power consumption, and network life. Habib et al. [16] proposed a protocol called evolutionary game-based routing (EGR) with multiple sinks for WMSNs. Here, evolutionary game theory (EGT) is also used in selecting cluster heads (CHs). In this EGR, the multimedia sensor nodes are provided with a data redundancy avoidance algorithm to reduce data redundancy based upon overlapping. This algorithm helps to improve network performance and energy efficiency by reducing the number of redundant transmissions. Therefore, this EGR technique provides better outcomes than other techniques.

Tripathi et al. [17] proposed an efficient multipath routing method for enhancing the lifetime of a WSN. An efficient multipath routing technique was focused on the residual energy and hop count to pick the multiple paths. The simplified energy design was utilized for data transmission. The experimental results showed that the routing protocol enhanced the performance of networks based on energy consumption, average end-to-end delay, first dead node, and packet delivery ratio. Due to more burst data, this technique was ineffective. Xu et al. [18] introduced the energy-efficient region source routing protocol (ER-SR) to maximize a WSN for a lifetime. The distributed energy region method was utilized for dynamically picking the nodes by higher residual energy. The effective distance-based ant colony optimization method was used for minimizing the consumption of energy for data transmission. The experimental results demonstrated that the scheme exhibited high efficiency and enhanced the performance on delivery delay. However, the network lifetime was affected because of high network overload.

Govindaraj et al. [19] presented the networks energy optimization for the internet of things (IoT) in a WSN by employing a capsule neural network (CNN) learning design. The neural network architectural design was employed for enhancing the network performance of the sensor and also the network optimization was executed, which was present within the wireless sensor network design and cloud storage space. The CNN design achieved higher performance by reducing the network energy in WSN. The computation time of this technique was high. To tackle the complications such as high packet loss and network congestion, Ambareesh et al. [20] suggested the hybrid red deer salp swarm (HRDSS)-based routing protocol in a WMSN. The experimental setup was examined using the NS2 simulator based on delay, memory, fitness value, and packet loss. The result illustrated that the HRDSS approach determines the optimal route for data transmission and minimizes packet loss, expected transmission (ETX) cost, and transmission delay efficiently; however, it failed to implement in engineering-related applications. Gutub et al. [21] discussed enhancing the image authentication process to secure the hidden data of watermarking in counting-based secret sharing. The newly generated data were shared with the embedded images with varied least significant bits of the pixels. As a result, the average capacity of the watermarking image authentication was achieved by 33–67%. However, the variation occurs in every size of the image and cannot adjust the location for securing the image. The creation of awareness to secure the prediction technology of the network using the simulated annealing algorithm and hybrid hierarchy genetic algorithm (SA-HHGA) based on the radial basis function (RBF) was illustrated by Chen et al. [22]. The experimentation results revealed that the predicted value of the developed method was processed with 15 samples, and it attained a stable condition in a normal situation. Meanwhile, it did not generate a guarantee for security. Gope et al. [23] presented a method for securing the lightweight privacy data obtained in a smart city environment by radio frequency identification (RFID) technology. The developed method was used to transmit the captured data in different geographical locations securely. As a result, various resources-constrained IoT devices were obtained for improving security. However, if the server concedes the RFID tag, an attacker could be able to capture all the authorization without authentication. 

Wani et al. [24] elaborated to protect lightweight protocols from anomalies by using the SDN-based intrusion detection method. The developed method did not generate a difficult situation in the security of IoT devices. As a result, it was operated in a real-world environment, which enhanced its performance. However, it required modifying the entire network for further processing. Das et al. [25] reviewed a method to secure the unauthenticated usage of data from illegal access in the healthcare industry. Here the data were protected by generating a security password to restrict the data access without permission. As a result, the developed method enhanced the effectiveness and strength of the security.

Verma et al. [26] discussed securing the sensitive data in Caesar cipher by converting the plaintext data into ciphertext, which boosted the security high by revisiting the shift cipher method. The experimentation results revealed that it improved the security performance in decrypting the data. On the other hand, decrypted data was hacked easily. Sarkar et al. [27] developed a method to reduce the consumption of time while using the android application to access information. The developed android application acquired various details of the users while registering the license number and the required information was shared through the web service. As a result, it improved the bandwidth while sharing the information with the user. Meanwhile, it paved the way to hack information easily. Detailed literature work presented in the Table 1. 

## 3. System Design

This section elaborates the system model of the WMSN in which it consists of network and energy designs. The numerical expression of these models is explained in the following subsections. 

### 3.1. Network Model 

**Figure 1:**
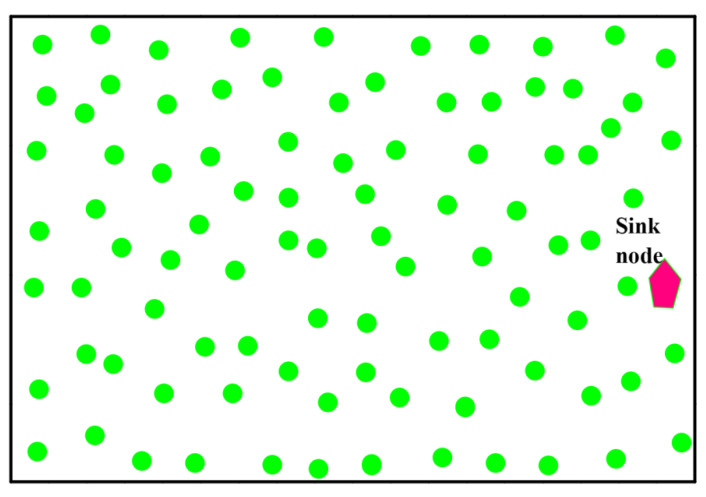
WMSN model.

Figure 1 depicts the WMSN containing n number of powered battery sensor nodes and base stations. WMSN is represented by a weighted undirected graph $g=\left(v_{s},l_{s},w_{s}\right)$, here the term $v_{s}=v_{1},v_{2},\ldots ,v_{n}$ depicts vertex set, $l_{s}=l_{1},l_{2},\ldots ,l_{n}$ indicates the set of bidirectional links and $w_{s}=w_{1},w_{2},\ldots ,w_{n}$ signifies the set of link weights [28]. The random deployment of SNs monitors and gathers the data from the network field. Furthermore, the collected data of SNs are stored in the BS for further processing. The sensors erected on the network field have a unique ID, a set of neighboring nodes with a communication radius (ℜ) that are delineated as $n\left(\nu_{x}\right)=\left\{\nu_{y}/D\left(\nu_{x},\nu_{y}\right)\leq ℜ\;\mathrm{and}\;\nu_{y}\in v_{s}\right\}$. The terms $n\left(\nu_{x}\right)$ and $D\left(\nu_{x},\nu_{y}\right)$ represent number of multimedia sensor nodes in the network and distance between the nodes $\nu_{x}$ and $\nu_{y}$, respectively. Due to the deployment of SNs in hostile regions, the battery replacement process becomes a complicated task. In this mechanism, the cluster member senses the data and transmits them to the CH. However, the WMSN is also subject to network hole issues which greatly affect the data transmission capability of network. Also, due to restricted resources such as network bandwidth, energy, and memory, the WMSN performance becomes extremely influenced. Therefore, to overcome these difficulties, the multipath routing mechanism is utilized. The parameters, namely, throughput, delay and data delivery ratio are the main consideration of this mechanism, which helps to detect the optimal path for transmitting the data to the BS. Thus, this technique enhances the performance of the WMSN in multiple aspects, particularly by increasing the lifespan and reducing energy consumption. Based on the QoS demands, the mechanism necessitates the data to flow through trouble free paths for delay sensitive and time critical data packets. 

### 3.2. Energy Model 

The battery-powered sensor nodes deplete their energy on continuous data transmission. Due to the deployment of SNs in the farthest location, it is difficult to recharge the batteries. Consider $E{\left(f,D\right)}_{t}$ as energy consumed by the SN to broadcast f-bit data to neighboring nodes and D implies the distance of two neighboring nodes. Each node senses data and broadcast to the neighboring nodes repeatedly, thereby depleting the energy of SNs. The energy consumption of SNs depends on the single-hop or multi-hop transmission. The mathematical expression for the computation of $E{\left(f,D\right)}_{t}$ is given by,

(1) $$E{\left(f,D\right)}_{t}=\left\{ \begin{array}{l} f.\;E_{e}+f.\;e_{Fl}.D^{2},D<D_{0}\\ f.\;E_{e}+f.\;e_{Ml}.D^{4},D\geq D_{0} \end{array}\right.$$

The term $E_{e}$ represents electronics energy, $e_{Fl}$ and $e_{Ml}$ indicates free space energy loss and multipath energy loss, respectively; $D^{2}$ and $D^{4}$ depicts power loss in single-hop and multi-hop transmission, respectively. Moreover, the threshold distance D0=eFleMl helps to find whether the data transmission is single-hop or multi-hop. The energy consumption according to transmission distance is computed as follows,

(2) $$E_{r}\left(f\right)=f.\;E_{e}$$

The above expression describes the energy consumption of SN to receive f data bits at distance D. 

### 3.3. Threat Model

Dolev-Yao (DY) is a well-known threat model which is applicable both in IoT as well as WSN environments for both wireless and wired sensor networks. According to the basics of DY design, two different communication groups can communicate over a public channel which is insecure. Various communication systems, namely, cloud servers, IoT sensors, and fog sensors are considered as non-trusted entities. 

Let us assume *A* be the adversary that modifies, deletes as well as inserts messages in or from the ongoing communication. In addition to this, *A* is capable of capturing few IoT sensors and extracts the most vital data from the memory. Also, *A* clones various new malicious nodes containing diverse attacking functionalities (i.e., wormhole, blackhole as well as sinkhole) along with the utilization of extracted information. Under execution of malicious attack, the data packets were dropped, lost, or denied, which degrades the performances of the communication.

## 4. Proposed Methodology

The numerous standard image compression methods are not properly suited for resource constrained but the transmission and image coding method can eliminate the resource constraint problem in WMSNs [29]. The significant intention of this paper is to eliminate the data redundancy and to select optimal cluster heads, thereby minimizing the energy consumption as well as enhancing the network lifetime [30,31]. Therefore, this paper proposes a novel FCSEO algorithm for optimal selection of CHs. The following subsection deliberates the workflow of the proposed methodology. 

### 4.1. Cluster Head Selection 

Various approaches have been used to enhance a specific metaheuristic technique and based on this process we propose a novel FCSEO algorithm. The FCSEO algorithm is capable of selecting an optimal cluster head with high network lifetime and minimum energy consumption. The following subsection delineates the steps involved in the FCESO algorithm-based cluster head selection process. 

#### 4.1.1. Criminal Search Optimization Algorithm (CSOA)

The objective of this criminal search optimization algorithm (CSOA) [32] is to find the criminal by the evidence that is collected by the investigators. Cooperative jamming and power randomness are used to improve covertness performance [33]. To achieve the goal of CSOA, the entire process is segmented into four phases. These phases are explained below:

Phase 1: Initialization

This initialization process is carried out in this phase. Here the parameters, SI (sub-investigators), and the undercover officers are initialized that are being used by the CSOA. At first, all informants and SIs are obtaining suspects’ random information at their pre-defined search location. The parameters such as $inq_{\max}$ and $Random_{\max}$ represent maximum inquiries and the random numbers’ maximum limit, respectively.

Phase 2: Evaluating the Performance and Searching for the Criminal

The chief investigator (CI) collects all the information that is gathered by the informer and SI and creates the evidence against the accused person by using the objective function. Initially (s=0), the defender is considered as primary suspect based on best fitness (evidence) as shown by below equation.

(3) $$P^{s}=B[SI^{s^{*}},In^{s^{*}}]$$

The terms $P^{s}$ is the primary suspects’ information that is available in CI; *B* represents the best, $SI^{s^{*}}$ represents the accused persons’ best information available with the *SI*; and $In^{s^{*}}$ represents the accused persons’ best information available with the informers; then s is for investigation steps. In the following phases (s≠0), the comparison is made between the primary accused persons’ present inquiry stage and previous inquiry stage to select the evidence of the present primary accused person by using Equation (4),

(4) $$BInfo^{s}=\left\{ \begin{array}{l} pri_{AP}^{s}\;\;\;\;\;\;\;\;if\;e(pri_{AP}^{s})<e(pri_{AP}^{s-1})\\ pri_{AP}^{s-1}\;\;\;\;\;\;\;\;else\;if\;e(pri_{AP}^{s})\geq e(pri_{AP}^{s-1}) \end{array}\right.$$

In the above equation, the term $BInfo^{s}$ represents the accused persons’ best information that is present with the CI; the term $pri_{AP}^{s-1}\;$ signifies the best prior information of the primary accused person with the CI; the terms $e(pri_{AP}^{s})$ and $e(pri_{AP}^{s-1})$ represents the evidence after processing $pri_{AP}^{s}$ and $pri_{AP}^{s-1}$ information, respectively; in the beginning, viz. when s=0, $pri_{AP}^{s}$ is considered as $BInfo^{s}$.

Phase 3: Update Information 

The updating of information is carried out in three steps. They are:

*Interrogation*—The accused persons’ information with the investigator is upgraded using Equation (5).

(5) $$BInfo^{s+1}=BInfo^{s}+BInfo^{s}\times R^{s}$$

From the above equation, the term $BInfo^{s+1}$ represent the primary accused persons’ updated information; the term $BInfo^{s}$ represents the primary accused persons’ present information which is available with CI; $R^{s}$ is the random number with the range [−1, 1]. The term s is the investigation stages.

*Information Updating of SI—*The information from *SI* is upgraded by using Equation (6) in this step.

(6) $$SI^{s+1}=SI^{s}+d_{1}\times SI_{BInfo}^{s}+x\times d_{2}\times SI_{y}^{s}+(1-x)\times d_{3}\times SI_{d}^{s}$$

The term $SI^{s}$ is the primary suspects’ present information that is available in *SI*; the term $SI^{s+1}$ is the primary suspects’ updated information that is available in *SI*; the evidence gathered from the primary accused person by the *SI* is represented as $SI_{BInfo}^{s}$ and is given as ($BInfo^{s}-SI^{s}$). The evidence gathered from previous best evidence by *SI* is given as ($SI_{BInfo}^{s-1}-SI^{s}$) is represented by $SI_{y}^{s}$. The evidence collected from random *SI* is given as ($SI_{d}^{s}-SI^{s}$) is represented by $SI_{d}^{s}$. The random numbers from [0, $d_{\max}$] range are represented as $d_{1},d_{2},\;\mathrm{and}\;d_{3}$ and $d_{\max}$ is a random number with a maximum limit. Here *x* is an influencing factor with [0, 1] range random number which represents *SI*’s influence over evidence after comparing.

*Information updating of Informer—*The information from informer is upgraded by using Equation (7) in this step.

(7) $$I^{s+1}=I^{s}+d_{1}\times I_{y}^{s}+d_{2}\times I_{SI}^{s}$$

The term $I^{s}$ define the accused persons’ evidence available with informers, the updated defender evidence present with the undercover officer is represented as $I^{s+1}$. Then the gathered evidence from previous evidence which is present with the undercover officer is shown as $\left(I_{y}^{s-1}-I^{s}\right)$ which is represented as $I_{y}^{s}$. The updated evidence gathered by an undercover officer from *SI* is represented as $I_{SI}^{s}$ and it is given as ($SI^{s+1}-I^{s}$).

Phase 4: Termination

After the information are updated from *SI* and informers, the updated data is reviewed to see if it is contained inside the search space. When the termination criteria ($s=s_{\max}$) is met, the inquiry is terminated and the primary accused person present is considered as criminal; otherwise, phases 2–4 are repeated. 

Phase 5: Output

The final phase of CSOA is the output phase, here the culprit is announced. When the requirement of termination is reached the inquiry is terminated. The criminal informer is considered as suspect information that generates the strongest proof, and the accused person is declared as the criminal. 

#### 4.1.2. Ebola Optimization Search Algorithm (EOSA)

This section represents the different stages of the condition checking and initialization process [30]. The computation process leading to the investigation and utilization of the EOSA algorithm is shown and the subgroup’s updated procedures are determined. The below equation which describes updating the positions of the individual is expressed as,

(8) $$Mi_{j}^{T+1}=Mi_{j}^{T}+\alpha N\left(i\right)$$

where α, N(i) represents the scale factor and movement rate, respectively. $Mi_{j}^{T}$, $Mi_{j}^{T+1}$ are the original and updated positions. The movement rate is defined as,

(9) $$N\left(i\right)=SRate*Rand\left(0,1\right)+N\left(ind_{Best}\right)$$

(10) $$N\left(S\right)=LRate*Rand\left(0,1\right)+N\left(ind_{Best}\right)$$

The term SRate implies short-distance movement. The investigation stage is used to find the impact when the infected individual shifts beyond the range of LRate. The LRate and SRate are arranged by using neighborhood parameters. 

Initializing the susceptible populations

Initially, the initial populations are generated by using a random value of distributions along with zero position. The generation of individuals is shown in the below equation,

(11) $$individual_{j}=l_{j}+Rand\left(0,1\right)*\left(u_{j}+l_{j}\right)$$

where,$u_{j}$, $l_{j}$ signifies both upper and lower boundaries, respectively. i represents the values from 1, 2, 3,……, n. The combination of best solution selection and infected individuals are shown in the below equation,

(12) $$BestD=\left\{ \begin{array}{l} G\;best,\;\;Fitness\left(C\;best\right)<Fitness\left(G\;best\right)\\ C\;best,\;\;Fitness\left(C\;best\right)\geq Fitness\left(G\;best\right) \end{array}\right.$$

Here, BestD, G best, C best represents the best solutions, global best solutions, and current best solutions respectively. G best, C best denote super-spreader and transmitter, respectively.

The vaccinated (v), infected (i), exposed (e), susceptible (D), hospitalized (h), quarantine (q), funeral (f), dead (d), and recovered (r) are used in the differential calculus and is shown below,

(13) $$\frac{\partial D\left(T\right)}{\partial T}=\pi -\left(\eta_{1}\;i+\eta_{3}\;d+\eta_{4}\;r+\eta_{2}\;\left(pe\right)\right)\;D-\left(\phi \;D+\varphi \;i\right)$$

(14) $$\frac{\partial i\left(T\right)}{\partial T}=\left(\eta_{1}\;i+\eta_{3}\;d+\eta_{4}\;r+\eta_{2}\;\left(pe\right)\;\psi \right)\;D-\left(\varphi +\theta \right)\;i-\left(\phi \right)\;D$$

(15) $$\frac{\partial h\left(T\right)}{\partial T}=\beta \;i-\left(\theta +\mu \right)\;h$$

(16) $$\frac{\partial r\left(T\right)}{\partial T}=\theta \;i-\varphi \;r$$

(17) $$\frac{\partial v\left(T\right)}{\partial T}=\theta \;i-\left(\lambda +\sigma \right)\;v$$

(18) $$\frac{\partial d\left(T\right)}{\partial T}=\left(\phi \;D+\varphi \;i\right)-\tau \;d$$

(19) $$\frac{\partial q\left(T\right)}{\partial T}=\left(\pi \;i-\left(\theta \;\;r+\varphi \;d\right)\right)-\omega \;q$$

The above-mentioned equations are denoted as a scalar function which has one value; that value is represented as a float. The exponential growth is described by using a scalar differential equation:

(20) $$k^{\prime }=\vartheta \;k$$

where, k implies the growth rate. 

#### 4.1.3. Formulation of Fuzzy Criminal Search Ebola Optimization (FCSEO)

**Figure 2:**
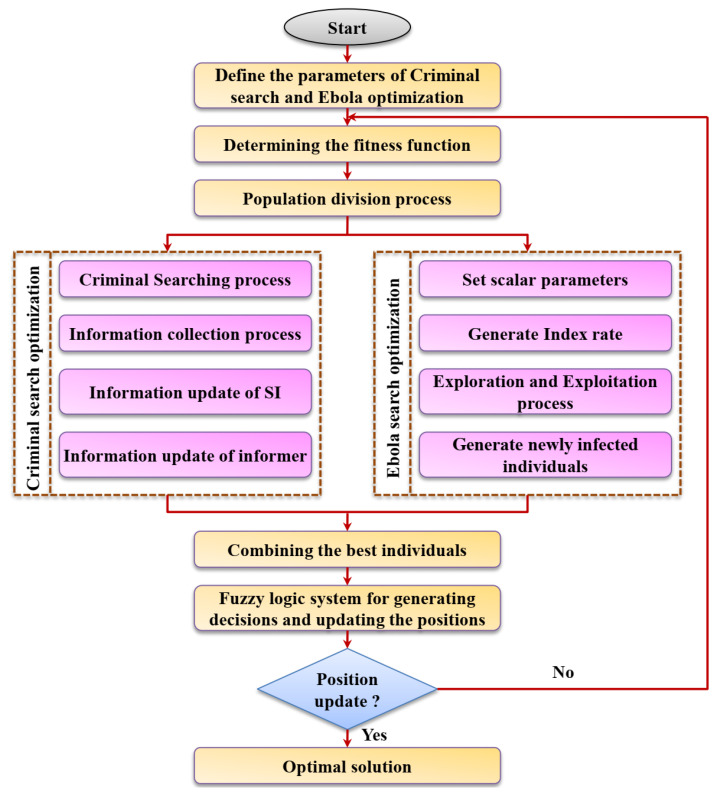
Formulation of the FCSEO algorithm.

➢ The Criminal search and Ebola optimization are novel algorithms which apply unique patterns for both exploration and exploitation. Both algorithms have their own advantages in solving the non-constrained statistical functions, but in the case of complex engineering problems, their performance often drops.
➢ The main reason is the exploration–exploitation tradeoff achieved via a constant ratio (neighborhood parameter or best information of the prime suspect). Often the best individual in both the algorithm populations is the individual with the maximum fitness which often becomes trapped in the local minimum.
➢ This is the major reason for premature convergence. These are the problems that affect the algorithm’s convergence rate and accuracy, and it is overcome in this work using the FCSEO algorithm. The exploration and exploitation behaviors are controlled here using the fuzzy decision-making strategy.
➢ A new support agent called a virtual individual is defined and included in the navigation process. The search behavior of the algorithm is constantly modeled using the fuzzy strategy based on a threshold value.
➢ The search procedure (optimal cluster head selection and energy minimization) is improved by applying a nine-rule fuzzy policy and speeding up the convergence.
➢ A virtual agent is introduced in the Ebola population to share every individual’s experience and also provide a different search direction for the population in each iteration.
➢ In this way, the individual in the population escapes the local minima and identifies some significant region in the search space [31]. The FCSEO algorithm gives equal chances to the individuals in the population to perform both exploration and exploitation in every iteration. Hence the CSO algorithm is used for local search and the Ebola algorithm is used for global search.
➢ The virtual agent (*B^V^*) is mainly induced inside the population instead of using the random interaction between the individuals in the population. According to the fitness function value, the direction of the individual in the population connected with the virtual agent is determined.
➢ The new self-adaptive method formulated is known as the FCSEO algorithm where the search behavior is controlled using a fuzzy decision module.
➢ The working of the FCSEO algorithm is shown in Figure 2 and the algorithm is formulated as shown below:

(21) $$B_{j}^{i+1}=B_{{}_{j}}^{i}+\partial .\psi_{j}.\left(z_{1}^{2}\times G^{*}-B_{{}_{j}}^{i}\right)+\beta .\bar{\partial }.\psi_{j}(z_{2}^{2}\times B_{{}_{j}}^{A(i)}-B_{{}_{j}}^{i})$$

Here, ${\bar{\partial }}_{i}$ is given as $1-\partial_{i}$ and

(22) $$\beta =\{\begin{array}{c} 1\;\;\;\;\;\;\;\;\;if\;f\left(B_{{}_{j}}^{A(i)}\right)<f(B_{{}_{j}}^{i})\\ -1\;\;\;\;\;\;\;\;if\;f(B_{{}_{j}}^{A(i)})\geq f(B_{{}_{j}}^{i}) \end{array}$$

The *j*th individual in the population is represented as the *j*th subscript, ∂ is the fuzzy decision factor, ${\bar{\partial }}_{i}$ is the complementary value of ∂, *z*_1_ and *z*_2_ are the two random numbers in the interval [0, 1], $\psi_{j}$ is the growth rate in the Ebola optimization algorithm, and $B_{{}_{j}}^{A(i)}$ is the virtual agent used. The fuzzy tendency factor is mainly used to alter the search behavior of the Ebola and criminal search optimization algorithms. The nine fuzzy rules used in the proposed work illustrate the search behavior of the individual in the algorithm. A normalized objective function called *G* is introduced to ensure fairness in this algorithm and the *G* function for the *j*th individual in the population is stated below:

(23) $$G_{j}=\frac{f(B_{j})-f(G*)}{f(B_{worst})-f(G*)+\tau }$$

In Equation (23), the *j*th object in the population is represented as $B_{j}$, the worst individual in the population is represented as $B_{worst}$, and the best solution is represented as G*. A positive scalar value called τ is used to control the zero-condition divergence. An energy-efficient route has a high $G_{j}$ value and the fuzzy tendency factor (G*) is obtained using the below equation.

(24) $$\partial_{o}=\partial_{o}^{old}+\Delta \partial_{o}$$

In Equation (24), $\partial_{o}^{old}$ is the previous fuzzy decision factor and the incremented fuzzy decision factor is represented as $\Delta \partial_{o}$. 

#### 4.1.4. FCSEO Algorithm for CH Selection

**Figure 3:**
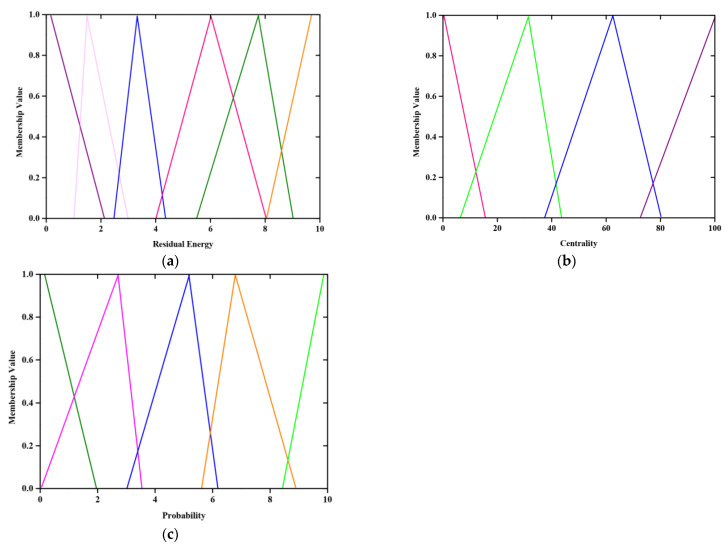
(**a**) fuzzy set for residual energy, (**b**) fuzzy set for centrality and (**c**) fuzzy set for provability of CH selection.

Residual energy (G) is considered as the main parameter of WMSN which decides whether the SN is to be selected as CH or hop nodes. In a fuzzy system, the linguistic variables namely Very Higher (*VH*), Higher (*H*), Medium (*M*), Lower (*L*) as well as Very Lower (*VL*) are introduced to delineate the residual energy. Figure 3 describes the fuzzy rule of residual energy (input variable). Moreover, radio energy dissipation is also considered to be a significant parameter for CH selection. Centrality $\left(\partial_{j}\right)$, is a parameter that calculates how central and the role of SN inside the cluster. The SN, which is located at center of cluster, has more possibility to be selected as CH. So, the central location of cluster is determined by the following expressions.

(25) $$C_{enter}A=a+\frac{w}{2}$$

(26) $$C_{enter}B=b+\frac{h}{2}$$

The term w indicates the width of cluster and h depicts height of cluster. Also, (a,b) represent the left down corner position of the cluster. Then the location of the cluster center is found by measuring the centrality of SN using Euclidean distance, which is given by,

(27) $$D\left(P_{ja},\;Cluster_{center}\right)=\sqrt{{\left(P_{ja}-C_{enter}A\right)}^{2}+{\left(P_{jb}-C_{enter}B\right)}^{2}}$$

From the above equation, Ath and Bth positions of SN are denoted as $P_{ja}$ and $P_{jb}$. Table 2 presents the 9 fuzzy mapping rules for the selection of CH. 

The FCSEO parameters G and $\partial_{j}$ represent the input variables residual energy and centrality; the parameter $\Delta \partial_{j}$ represents the output variable probability of CH selection.

Figure 3a illustrates the fuzzy rule for generating residual energy (input variable) where purple color signifies the membership function with very high value (VH), blue indicates higher value (H), Pink represents medium value (M), Green and Orange represents low (L) and very low value (VL). 

Figure 3b indicates the fuzzy rule generation based on centrality. Pink and Green lines denote very high (VH) and high (H) values, Blue and purple represent Medium (M) and low values (L) respectively. 

Figure 3c portrays the probability of selecting the cluster head; where green and pink signifies very high (VH) and high values (H), blue indicates medium value(M), orange and green represents low (L) and very low (VL)values respectively. 

### 4.2. Data Redundancy Process 

When multimedia sensors are placed close to each other, an overlapped field of view (FoV) is determined. The threshold value *τ* mainly denotes the part of the isosceles triangle in FoV and M represents the congruent side of the triangle. If any item is present in between the FoV of two interlinked sensor nodes, then it results in a polygon. The polygon mainly implies that the data is redundant if two nodes send the image of the item. The shapes such as quadrilateral and triangle are not always large enough to fit inside the threshold or minimal threshold. Since the triangle and quadrilateral generated can be small or big, the data redundancy algorithm used in our paper is also formulated using the same concept. The data redundancy algorithm is specified in Algorithm 1 and the object that overlaps in the FOV regions is said to be redundant. The nodes with the high energy elected as the cluster head by the FCSEO optimization algorithm are used to transmit the data.
**Algorithm 1:** Data redundancy modelWMSN node-1→a1, WMSN node-2→a2, Remaining energy of a1→z1, Remaining energy of a2→z2 Initialize τ confirm whether the Euclidean distance (EC) between a1 and a2 is EC > 2 M If (EC > 2 M)  No overlap found in the FOV End If If (EC <= 2 M)  If the shape is a polygon, then     Redundancy is present  End If    If shape is either quadrilateral or triangle      Compute the area of either the quadrilateral or triangle    End If   If (Area > τ)        Redundancy present   Else        Redundancy absent   End If End If If (z1 > z2)  Then a1 is responsible for the items FoV and the CH transmits the data Else Then a2 is responsible for the items FoV and the CH transmits the data End If

## 5. Experimental Results and Discussions

The experimental analysis is carried out under MATLAB 2018 (b) version containing an internal processor of Intel^®^ Core TM i7-3770 CPU@3.40 GHz, 500 Gb, 12 GB RAM for simulation. By comparing with some existing methods, the efficiency of the proposed technique is determined. The MATLAB is employed, which includes timings, protocols, nodes, etc. The simulation parameters are mentioned in Table 3. 

### 5.1. Parameter Description

The parameters utilized to tune the proposed method are represented in Table 4.

### 5.2. Evaluation Measures

#### 5.2.1. Network Lifetime analysis ($N_{l}$)

The ratio of the active node ($A_{Node}$) to the availed node ($T_{Node}$) is referred to as network lifetime.

(28) $$N_{l}=\frac{A_{Node}}{T_{Node}}$$

#### 5.2.2. Packet Delivery Ratio ($PD_{ratio}$)

The fraction of delivered packet to the transmitted packet is termed as the packet delivery ratio.

(29) $$PD_{ratio}=\frac{Total\;\;number\;\;of\;delivered\;packets\;\;}{Total\;number\;of\;transmitted\;packets\;\;}\;$$

#### 5.2.3. Throughput

The total time taken for the amount of data to transmit or receive to the destination node is known as throughput.

(30) Tp = Amount  of data to transmit or receive Required time 

#### 5.2.4. End-to-End Delay

The mathematical relation of delay is expressed in Equation (31),

(31) $$\mathrm{END}-{\mathrm{END}}_{delay}=\;\frac{\sum_{k=0}^{q}\mathrm{END}-{\mathrm{END}}_{\partial }}{Number\;of\;data\;\;transmitte\;d}\;$$

In the above equation, the packet id is represented as q and the number of packets received is represented as k .

#### 5.2.5. Residual Energy 

After each data transmission and sensing, some amount of energy is lost in the SNs and the remaining energy availed in the SNs is termed as residual energy. The mathematical expression for $Average\;\lambda_{\varepsilon }$ is given as,

(32) $$Average\;\lambda_{\varepsilon }=\frac{1}{q}\sum_{z\in q}\lambda_{\varepsilon }^{z}$$

The term $\lambda_{\varepsilon }^{z}$ represents the residual energy availed in the SNs and the amount of sensor node is denoted q. 

#### 5.2.6. Energy Consumption 

The amount of energy consumed in each number of rounds by the sensor node is termed as energy consumption. Equation (33) represents the standard deviation of energy consumption:

(33) φec=1q∑z=1q(Average λε−Jconsumed(z))2 

From the Equation (33), the number of alive nodes is represented as q, the average residual energy is $Average\;\lambda_{\varepsilon }$, energy consumed by zth  sensor is denoted as $J_{consumed}$.

#### 5.2.7. Structural Similarity Index Measure (SSIM)

The mathematical relation of SSIM is expressed in Equation (34),

(34) $$SSIM\left(a,b\right)=\frac{\left(2\;\sigma_{a}\;\sigma_{b}\;+D_{1}\right)\;\left(2\;\alpha_{ab}+D_{2}\right)}{\left(\sigma_{a}^{2}+\sigma_{b}^{2}+D_{1}\right)\;\left(\alpha_{a}^{2}+\alpha_{b}^{2}+D_{2}\right)}$$

#### 5.2.8. Routing Overhead 

The control overhead mainly refers to the transfer of the control information such as channel length, queue length, to the overall information acquired at each sensor node. 

### 5.3. Performance Evaluation 

**Figure 4:**
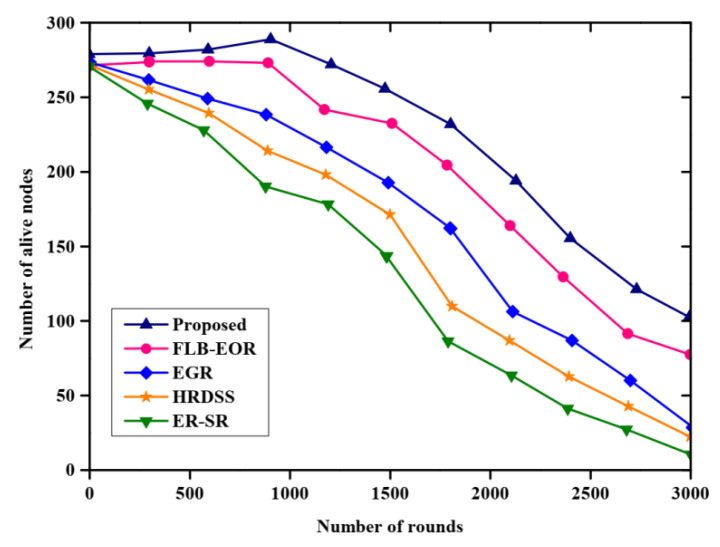
Comparative analysis of network lifetime.

Figure 4 shows the network lifetime analysis of the proposed FCSEO method. The FCSEO method is compared with various protocol techniques, namely, the efficient region source routing (ER-SR) protocol, firefly load balancing based energy optimized routing(FLB-EOR), evolutionary-game-based routing (EGR), hybrid red deer salp swarm (HRDSS). The proposed method has attained maximum alive sensor nodes compared to ER-SR, FLB-EOR, EGR, and HRDSS. The number of alive nodes in each round is greater for the proposed FCSEO method, with 275 alive nodes in 10,000 rounds, 225 alive nodes in 2000 rounds and 100 alive nodes in 3000 rounds, while for other compared methods the values are lower than the proposed FCSEO method. 

**Figure 5:**
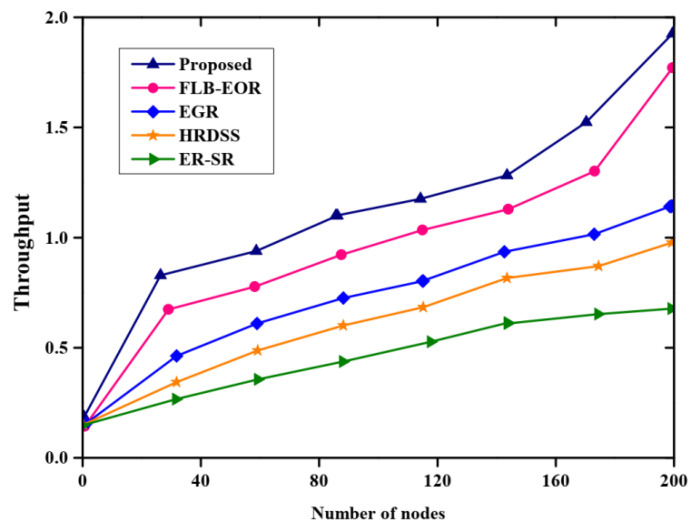
Comparative analysis of throughput.

Figure 5 and Table 5 depicts the analysis of throughput with respect to the number of nodes. The throughput of the proposed method was compared with the throughput of some existing methods such as ER-SR, FLB-EOR, EGR, and HRDSS. From the analysis, the proposed method obtains higher throughput than other methods. The royal blue line graph denotes the proposed method. The throughput value obtained by the proposed FCSEO method is high as compared to other methods with the value 0.75 for 40 nodes, 1.1 for 120 nodes and 1.9 for 200 nodes. 

**Figure 6:**
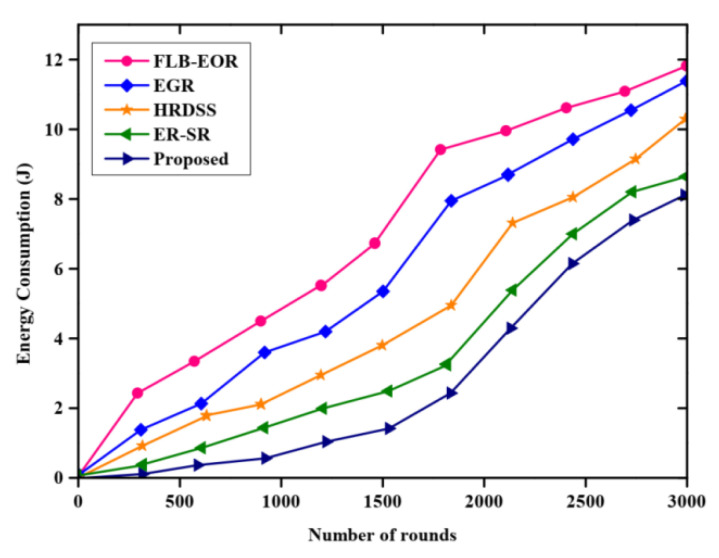
Comparative results of energy consumption.

Figure 6 describes the comparative evaluation of the energy consumption of various methods. The graph is plotted with the number of rounds and energy consumption. The proposed method is compared with various existing methods such as ER-SR, FLB-EOR, EGR, and HRDSS. Lower consumption of energy in the proposed approach maximizes the network lifetime. The energy consumed by the nodes in each round is less for the proposed FCSEO method than other compared methods. For the proposed FCSEO method, the energy consumed in 1000 rounds is 0.2 J, 2000 rounds is 3.5 J, and 8 J for 3000 rounds. 

**Figure 7:**
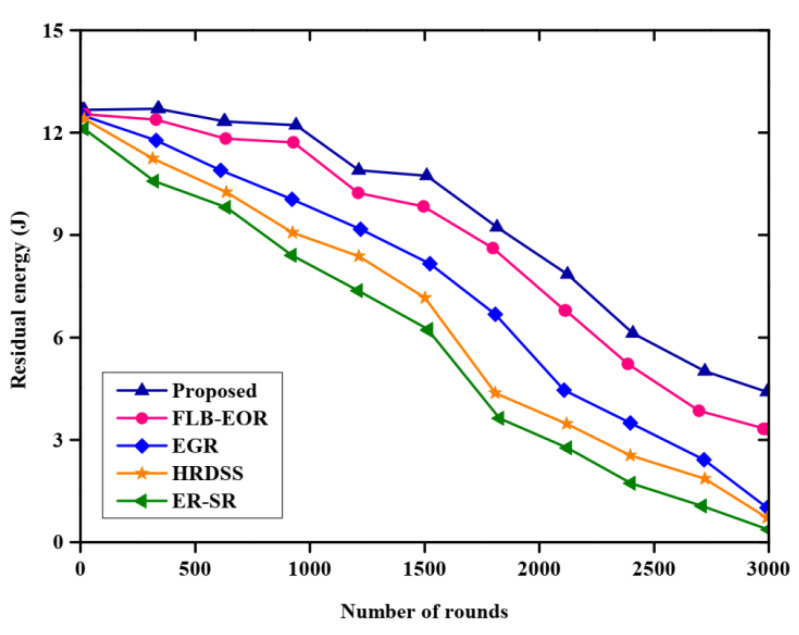
Comparative analysis of residual energy.

Figure 7 and Table 6 portray the comparative analysis of residual energy, and the proposed approach is compared with various existing methods, such as ER-SR, FLB-EOR, EGR, and HRDSS. More energy is stored in the proposed method than other methods, however, at a particular number of rounds, there is a drop in energy. The proposed FCSEO method achieves greater residual energy as compared to other with 12 J in 1000 rounds, 9 J in 2000 rounds and 5.9 J in 3000 rounds, while for other techniques the value is lower. 

**Figure 8:**
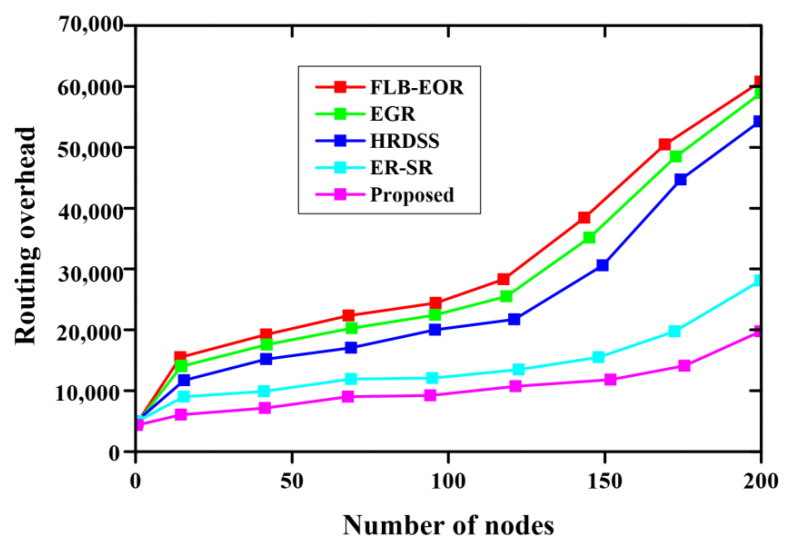
Comparative analysis of routing overhead.

**Figure 9:**
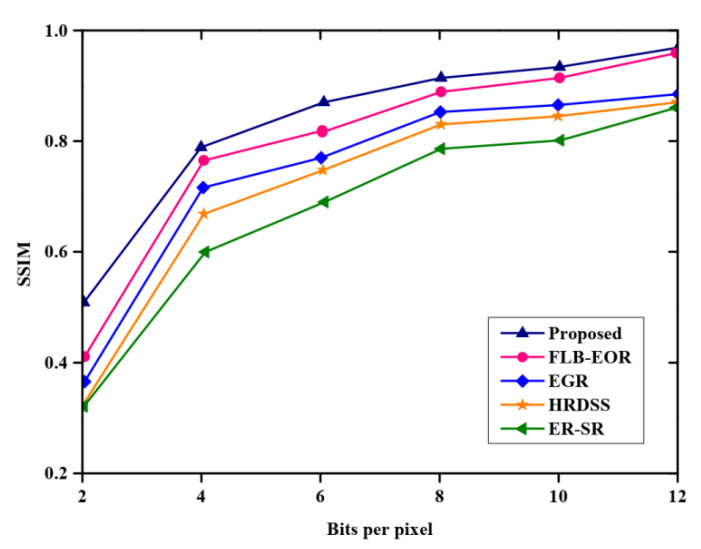
Comparative analysis of SSIM.

Figure 8 and Table 7 indicate a comparison of the routing overhead of the proposed method, where the *x*-axis specifies the nodes, and the *y*-axis specifies the routing overhead. From the figure, the proposed method has a lower routing overhead than ER-SR, FLB-EOR, EGR, and HRDSS. The overhead value increases for each number of nodes. The royal blue graph line represents the proposed method. With the increasing number of nodes, the overhead increases. The proposed FCSEO method achieves less routing overhead than other compared methods, achieving 5500 for 50 nodes, 5700 for 100 nodes, 5900 for 150 nodes, and 10,150 for 200 nodes. Figure 9 depicts the comparative analysis of the structural similarity index measure (SSM) of the proposed method. The proposed method is compared with various existing methods, such as ER-SR, FLB-EOR, EGR, and HRDSS. A higher structural similarity index measure was achieved by the proposed method. For 6 bits per pixels, the SSIM value obtained is 0.87, and for 12 bits per pixel, the SSIM value achieved is 0.96 by the proposed FCSEO method. 

The comparative analysis of the packet delivery ratio is depicted in Table 8. Here, *x*-axis specifies the nodal number, and the *y*-axis specifies the packet delivery ratio. The proposed method is compared with various existing methods, such as ER-SR, FLB-EOR, EGR and HRDSS. The proposed method achieves a high packet delivery ratio than other methods. 

Table 9 represents the determination of end-to-end delay, where the *x*-axis specifies the nodes and the *y*-axis specifies the end-to-end delay in seconds. The efficiency of the proposed approach was evaluated by comparing with existing methods, such as ER-SR, FLB-EOR, EGR and HRDSS. The end-to-end delay of the proposed technique is very low compared to other methods. 

## 6. Conclusions

This paper proposed a novel fuzzy criminal search Ebola optimization (FCSEO) algorithm for optimal selection of cluster heads. The significant intention of this paper is to eliminate data redundancy and to select optimal cluster heads thereby minimizing the energy consumption as well as enhancing the network lifetime. Also, the data redundancy present in the proposed algorithm is mitigated and thus the network lifetime is enhanced. The experimental setup was executed using the MATLAB platform by evaluating various parameters. Finally, the proposed method is compared with efficient region source routing protocol, firefly load balancing based energy optimized routing, evolutionary-game-based routing, and hybrid red deer salp swam. From the extensive experimentation, the analysis revealed that the proposed approach attains higher performances than the existing technique. The throughput value obtained by the proposed FCSEO method is high compared to other methods, with the value 0.75 for 40 nodes, 1.1 for 120 nodes, and 1.9 for 200 nodes. With the increasing number of nodes, the overhead increases. The proposed FCSEO method achieves lower routing overhead than other compared methods, achieving 5500 for 50 nodes, 5700 for 100 nodes, 5900 for 150 nodes, and 10,150 for 200 nodes. The proposed FCSEO method achieves greater residual energy as compared to others with 12.J in 1000 rounds, 9 J in 2000 rounds, and 5.9 J in 3000 rounds, while for other techniques the value is lower. In future, the proposed approach will be evaluated by including the real-time videos for data transmission as well as data aggregation.

## Figures and Tables

**Table 1:** Literary works of various authors.

Author and Year	Technique	Objective	Pros	Cons
Rehman et al. (2021) [11]	Sub-cluster head (SCH)	Improving resource constraints in IoT devices	Increased throughput and energy consumption	Low-speed applications
Aswale et al. (2021) [12]	Geographic multipath routing	Eliminate hidden node problem	Improved performance and network lifetime	Cost rate was high
Genta et al. (2019) [13]	Energy-effective multipath routing (EMR)	Decrease energy consumption while communicating	High performance, minimum energy consumption	Not suited for mobile sensor nodes
Raja et al. (2019) [14]	Firefly load balancing based energy optimized routing (FLB-EOR)	Minimum weightage for multimedia data transmission	Enhanced LBE and throughput	Energy consumption was higher
Awad et al. (2018) [15]	Gaussian distribution framework	Multipath routing for delay node optimization	Improve packet delay, data distribution, power consumption, and network life	Transmission delay was higher
Habib et al. (2019) [16]	Evolutionary game-based routing (EGR)	Reduce data redundancy	Enhanced network performance and energy efficiency	Failed to apply in real time
Tripathi et al. (2021) [17]	Efficient multipath routing method	Enhance network lifetime	Improve network performance	Ineffective due to burst data
Xu et al. (2019) [18]	Efficient region source routing protocol (ER-SR)	Maximize the lifetime of WSN	High efficiency and enhanced the performance	High network overload
Govindaraj et al. (2020) [19]	Capsule neural network (CNN)	Enhancing the network performance of the sensor	Achieved higher performance	High Computation time
Ambareesh et al. (2021) [20]	Hybrid red deer salp swarm (HRDSS)	To tackle the complications such as high packet loss and network congestion	Reduce packet loss, Expected Transmission (ETX) cost, and transmission delay	Not implemented in engineering-related applications
Gutub et al. (2022) [21]	Image authentication model	Improve the image authentication process to secure the hidden data of watermarking	Improve performance	Not able to adjust the location
Chen et al. (2022) [22]	Simulated annealing algorithm and hybrid hierarchy genetic algorithm (SA-HHGA)	To secure private data	Attained stable condition	Security was not guaranteed
Gope et al. (2018) [23]	Radio frequency identification (RFID)	Secure the lightweight and privacy data obtained in a smart city	Improved security	Attacker could be easily hacked without authorization
Wani et al. (2021) [24]	SDN-based intrusion detection	To protect lightweight protocols from anomalies	Improved performance	Processing was further performed after modifying
Das et al. (2022) [25]	Lightweight and anonymous mutual authentication scheme	To secure the unauthenticated usage of data from illegal access	High performances	Improve effectiveness and strength of security
Verma et al. (2022) [26]	Shift cipher technique	Securing sensitive data	Improve security performance	decrypted data was easily hacked
Sarkar et al. (2015) [27]	Web service-based android application	Reduce the consumption of time while using the android application	Improved the bandwidth	Hacked information easily

**Table 2:** Fuzzy set for CH selection.

Sl.No.	Residual Energy (G)	$\mathrm{Centrality}\;(\partial_{j})$	$\mathrm{Probability}\;\mathrm{of}\;\mathrm{CH}\;\mathrm{Selection}\;(\Delta \partial_{j})$
Rule 1	*H*	*H*	*VH*
Rule 2	*H*	*M*	*H*
Rule 3	*H*	*L*	*M*
Rule 4	*M*	*H*	*M*
Rule 5	*M*	*M*	*L*
Rule 6	*M*	*L*	*L*
Rule 7	*L*	*H*	*L*
Rule 8	*L*	*M*	*VL*
Rule 9	*L*	*L*	*VL*

**Table 3:** Parameters employed for simulation.

Parameters	Values
Total area	100 × 100 m
Offset angle	60 degrees
Total number of sensor nodes	100
Data and frame rates	2 Mbps, 30 fps
Initial energy	2 joules
Size of an image	175 × 145

**Table 4:** Parameter description for proposed method.

Techniques	Parameters	Ranges
Ebola Optimization Search Algorithm	Population size	100
	Total number of iterations	50
	Contact rate of infectious individuals	0.1
	Hospitalization rate	[0, 1]
	Recovery rate of human individuals	
Criminal Search Optimization Algorithm	Population size	50
	Maximum number of sub investigators	25
	No. of informers	15
	Total no. of iteration	100
	Maximum limit of random number	2

**Table 5:** Comparative results based on throughput.

Total Number of Nodes	Methods				
	Proposed	FLB-EOR ( )	EGR ( )	HRDSS ( )	ER-SR ( )
0	0.17	0.16	0.16	0.16	0.16
40	0.75	0.6	0.4	0.15	0.23
80	1	0.8	0.48	0.49	0.34
120	1.1	0.9	0.57	0.51	0.4
160	1.3	1.1	0.63	0.68	0.45
200	1.9	1.7	0.9	0.7	0.47

**Table 6:** Comparative results based on residual energy.

Total Number of Rounds	Methods				
	Proposed	FLB-EOR	EGR	HRDSS	ER-SR
500	13	12.3	11.6	11.3	10.5
1000	12.7	12.1	9.4	9	8.9
1500	11.8	11	8.9	7	6.1
2000	9	8.5	5.7	4	3
2500	7	5.5.	4	2.8	1
3000	5.9	4	1	0.9	0.4

**Table 7:** Comparative results based on routing overhead.

Total Number of Nodes	Methods				
	Proposed	FLB-EOR	EGR	HRDSS	ER-SR
0	5000	5000	5000	5000	5000
50	5500	18,000	18,000	17,000	8000
100	5700	21,000	20,000	18,000	8200
150	5900	35,000	31,000	29,000	10,100
200	10,150	58,000	56,000	54,000	22,000

**Table 8:** Comparative results based on packet delivery ratio.

Total Number of Nodes	Methods				
	Proposed	FLB-EOR	EGR	HRDSS	ER-SR
0	0.9	0.89	0.89	0.88	0.87
50	0.85	0.78	0.76	0.75	0.72
100	0.82	0.77	0.67	0.63	0.59
150	0.69	0.49	0.48	0.44	0.38
200	0.5	0.38	0.36	0.33	0.29

**Table 9:** End-to-end delay analysis.

Total Number of Nodes	Methods				
	Proposed	FLB-EOR	EGR	HRDSS	ER-SR
0	0.87	0.92	1.3	2.33	1.54
50	0.88	0.97	1.45	3.49	2.63
100	1.2	1.7	1.97	4.03	4.27
150	1.4	1.9	2.23	6.37	6.68
200	1.7	2.1	2.4	8.49	8.67

## Data Availability

Not applicable.
